# Prevalence and predictors of undernutrition among underfive children in Arusha District, Tanzania

**DOI:** 10.1002/fsn3.798

**Published:** 2018-10-08

**Authors:** Dyness Kejo, Theobald C. E. Mosha, Pammla Petrucka, Haikael Martin, Martin E. Kimanya

**Affiliations:** ^1^ Nelson Mandela African Institution of Science and Technology (NM‐AIST) Arusha Tanzania; ^2^ Tanzania Agriculture Research Institute (TARI) Arusha Tanzania; ^3^ Sokoine University of Agriculture Morogoro Tanzania; ^4^ University of Saskatchewan College of Nursing Saskatoon Canada

**Keywords:** predictors, stunting, Tanzania, underweight, wasting

## Abstract

Childhood undernutrition is a global health challenge impacting child growth and survival rates. This deficit in nutritional status contributes to the increasing chronic disease prevalence and economic burden in individuals and throughout developing contexts. A community‐based cross‐sectional study was conducted in Arusha District of Tanzania to determine the prevalence and predictors of undernutrition in 436 children. A structured questionnaire was used to collect data on demographic and socio‐economic factors as well as feeding practices and prevalence of preventable childhood diseases. Anthropometric data were collected through the measurement of length/height and weight of all children. The prevalence of undernutrition was estimated based on *Z*‐scores indices below −2SD of the reference population for weight for age (underweight), height for age (stunting), and weight for height (wasting). Fifty percent, 28%, and 16.5% of the children were stunted, underweight, and wasted, respectively. The age above 2 years and being a male were associated with stunting. The age above 2 years, nonexclusive breastfeeding children, and living at Seliani and Oturumeti were associated with being underweight. Similarly, morbidity, none exclusively breastfed children, living at Oturumeti, and being born to a mother 35 years and above were associated with wasting. In this study, we found the prevalence of child undernutrition in Arusha District is high in comparison with national and regional trends and appears to be associated with being a male. It is recommended that nutritionists and health planners should focus on these key predictors when planning nutrition interventions to address the problem of undernutrition among underfive children in Arusha District.

## 1 INTRODUCTION

Childhood undernutrition is a global health challenge impacting child growth and survival rates, contributing to future increased chronic disease prevalence, and reducing individual and national economic productivity (Black et al., 2013; Chang, Walker, Grantham‐McGregor, & Powell, 2002; Srivastava, Mahmood, Srivastava, Shrotriya, & Kumar, 2012). Childhood undernutrition is embedded within the complexities of intersectoral and multilevel interfaces related to food security and equities in health. Inadequate access to the “basics” causes childhood undernutrition and is realized in the forms of inadequate nutrition due to improper child feeding and infectious diseases (i.e., diarrhea, acute respiratory infection, and malaria); lack of sanitation and clean water; and limited access to necessary and appropriate health care (Dangour et al., 2013; De Onis, Blössner, & Borghi, 2012) underpin this state. Childhood undernutrition has frequently been cited as an indicator to measure the development of a nation (Badake et al., 2014; Black et al., 2013) as it reflects how the most vulnerable is treated and is used as an indicator to measure progress toward the millennium development goals (MDG1) which aimed to halve the percentage of people who suffer from hunger by 2015.

Global efforts to positively address childhood undernutrition have been made but deficits and challenges persist. Monitoring indicator trends, such as wasting, underweight, and stunting, have dominated made discussions regarding progress in developing countries. Wasting, which is linked to acute undernutrition and manifests as a 10% or greater unwanted weight loss, is often associated with droughts and cyclical/seasonal periods of food insecurity (UNICEF, 2013). In 2014, the global prevalence of wasting was reported to be 7.5% (UNICEF, WHO, & World Bank, 2015). The prevalence of underweight declined from 25% to 16% (UNICEF, 2013). Stunting, which is linked to chronic undernutrition and manifests as short in stature for age cohort, is a widespread problem in many parts of the world (UNICEF, 2013). According to UNICEF (2013), height at 2 years has been reported as the strongest predictor of future human capital. Although global stunting rates have decreased from 39.6% in 1990 to 23% in 2016, the issue remains prevalent especially with males (Chirande et al., 2015; UNICEF, 2017) and is nearing endemic rates in African regions.

Nutritional interventions have been developed and implemented in Tanzania by the Ministry of Health (MOH) to reduce child undernutrition. These are infant and young child feeding (IYCF) protocols, sanitation, deworming, vitamin A supplementation, and health education (MOH, 2008). Despite these interventions, child undernutrition still remains a developmental challenge. According to the Tanzania Demographic Health Survey (TDHS) report, the national profile shows wasting prevalence of 5%, underweight at 14%, and stunting of 34% (National Bureau of Statistics (NBS) & MACRO, 2015). The prevalence of undernutrition in Tanzania is relatively well documented at the regional levels, but not specific at the district levels. Compared to other regions, Arusha Region reported a higher prevalence of wasting (6.5%), underweight (20%), stunting (36%), and anemia (57%) than the national rates (NBS & MACRO, 2015). This local trend was attributed to poor feeding practices and minimal consumption of fruits and vegetables (NBS & MACRO, 2010). In Arusha Region, reports on child nutritional status at district level are lacking. The only available reports are of studies focusing on the Maasai communities (Agho, Inder, Bowe, Jacobs, & Dibley, 2009; Nyaruhucha, Msuya, Mamiro, & Kerengi, 2006). This paper highlights the prevalence and predictors of undernutrition among underfives in Arusha District, a multiethnic locale within Arusha Region.

## 2 MATERIALS AND METHODS

### 2.1 Study area

The study was conducted in three wards of Arusha District, namely Oldonyosambu, Oturumeti, and Seliani. Children in rural areas are more likely to be more undernourished than their urban counterparts (National Bureau of Statistics & Macro, 2015); therefore, Arusha District, which is predominantly rural, was selected. Geographically, this district surrounds the Arusha Municipal and is economically rooted in agriculture and livestock production (United Republic of Tanzania [URT], 2014). Arusha District has 56,196 children under 5 years of age (28,397 females; 27,799 males) (National Bureau of Statistics & Macro, 2015). The district has two hospital, four health centers, and 23 dispensaries (URT, 2014).

### 2.2 Study design

A quantitative cross‐sectional community‐based survey was used in this study.

### 2.3 Subject inclusion criteria

The inclusion criteria used for subjects’ selection included family residence within the study villages, child (ren) between 6 and 59 months, and child (ren) consuming some solid foods (i.e., no longer exclusively breastfeeding). The exclusion criteria included child (ren) below 6 months (regardless of solid food intake), child (ren) aged above 60 months, or the family rejected to participate in the study.

### 2.4 Sampling procedure

The multistage sampling was used to select one district from seven districts in Arusha Region, whereby Arusha District (one of the districts in Arusha Region) was selected purposively. Arusha District has 20 wards which constituted the sampling frame. The sampling interval was calculated by dividing with the number of required clusters which was 3. Random numbers were generated using the computer method in order to obtain the required clusters with the first number produced by the computer yielding the first cluster with the subsequent cluster obtained by adding the cluster with sampling interval. In the selected wards, the research team with the help of village health workers visited all households with children aged 6–59 months. The purpose and nature of the study activities explained to parents/caregivers and those who agreed to participate gave consent and were invited to come to the nearby Reproductive and Child Health (RCH) care center.

### 2.5 Sample size calculation

The sample size was determined using the formula *n* = *z* ^2^ *p* (1−*p*)/*d* ^2^ (Burger & Pierre‐Louis, 2002). The formula was populated using the following: 44% of underfive children are stunted (*p*) with a 95% confidence interval and 5% marginal error (*d*). The calculated sample size was 378, with a final sample size of 436 mother–child dyads included allowing for an anticipated 15% declining to participate (Adu‐Afarwuah et al., 2007).

### 2.6 Construction of questionnaire, pretesting, and administration

A questionnaire was administered through face‐to‐face interview administered by a trained research assistant with the mothers/guardians. The data collection tool was pretested on a group of nonparticipating subjects, reflected upon, and validated accordingly. Standard techniques and equipment were used for collecting anthropometric measurements (De Onis, 2006) that reflected the variables of height, weight, sex, and age.

### 2.7 Measurements taken and tools

Anthropometric indicators used in this study aligned with international standards for underweight, stunting, and wasting. Undernutrition was determined if the child is either stunted, underweight, wasted, or a combination of two or more.

A Seca™ electronic scale was used for the weight measurement. The device was placed on a flat floor and standardized to zero at the start of each day. To measure a child who could not stand alone, the mother was asked to step on the scale, without the child, for the mother's weight to be displayed, and the scale was then tared (zero out) when the child was passed to the mother, thereby displaying the child's weight. In cases where the child was able to stand alone, the child was weighed directly. In all cases, the scale was read to the nearest 0.1 kg with minimal/light clothing and no shoes.

Recumbent length was measured for children under the age of 2 years. In these cases, the child was placed horizontally on a wooden measuring board (Perspective Enterprises™, Portage, MI, USA). The subject was placed facing upward with the head toward the fixed end and the body parallel to the long axis of the board. The subject's knees were pressed onto the board so that the legs were straight and the toes pointing directly upward, and then, the movable footboard was brought to rest firmly against the heels and measured to the nearest 0.1 cm (Gibson, 1990). Standing height for children above 24 months was measured vertically using a wooden measuring board (Perspective Enterprises™) measured to the nearest 0.1 cm (Badake et al., 2014). All the anthropometric measurements were taken by two nutritionists.

### 2.8 Data analysis

The data collected were entered, cleaned, coded, and analyzed using SPSS™ Version 20 Nutritional data were entered and analyzed using SMART™ software provided by the WHO. Chi‐squared tests were used to compare group differences for categorical variables. The dependent variables included were underweight, stunting, and wasting while the independent variables were child's demographic information (i.e., sex, age, morbidity information, and feeding practices) and parental socio‐demographic information (i.e., education level, age, and marital status). Bivariate logistic regression was performed to control for confounders for three outcome variables: underweight, stunting, and wasting separately. The independent variables found significant (*p*‐value equal or less than 0.05) during bivariate analysis were selected for multivariable analysis. Multivariable binary logistic regressions were fitted using the backward elimination technique to identify determinants of underweight, stunting, and wasting separately. The association between dependent and independent variables was assessed using odds ratio (OR), adjusted odds ratio (AOR), and 95% confidence interval (CI). The statistical association was declared significant if the *p*‐value was less than 0.05.

### 2.9 Ethical clearance

Ethical clearance was obtained from the Ethics Committee of the National Institute of Medical Research (NIMR) in Tanzania. Permission to undertake the study in Arusha District was obtained from the regional and district health departments.

## 3 RESULTS

A total of 436 children aged 6–59 months participated in the study. Table 1 summarizes the socio‐demographic characteristics.

**Table 1:** Socio‐demographic characteristics of participants by gender

Variable	*N*	Female (%)	Male (%)	*p*
Residence				
Oldonyosambu	159	41.8	32.2	0.06
Seliani	150	34.0	37.7	
Oturumeti	127	24.2	33.1	
Mother's age (years)				
15–24	213	53.6	45.0	0.4
25–34	197	43.3	46.7	
35–49	26	3.1	8.3	
Mother's education				
No formal education	*67*	12.4	17.8	0.2
Primary education	*291*	67.0	66.5	
Secondary and above	*78*	20.6	15.7	
Father's education				
None or primary incomplete	36	7.7	8.7	0.8
Primary complete	347	80.9	78.5	
Secondary and above	53	11.3	12.8	
Marital status				
Single	*44*	9.8	10.3	0.8
Married	*392*	90.2	89.7	
Child's age (months)				
6–24	291	63.4	69.4	0.2
Above 24	145	36.6	30.6	
Morbidity				
No	142	34.5	31.0	0.4
Yes	294	65.5	69.0	
Types of diseases				
Diarrhea	66	23.4	22.0	0.7
Fever	51	17.7	17.3	
Cough	25	6.5	10.1	
Multiple diseases	150	52.4	50.6	
Family size				
<5	303	71.1	68.2	0.5
Above 5	133	28.9	31.8	
Income level (Tshs)				
>2,500	59	15.5	12.0	0.3
<2,500	377	84.5	88.0	

Number of children *p* value significant at <0.05 (in italics).

### 3.1 Child feeding practices

Table 2 offers a comparison of feeding practices. There was an apparent gender bias in the practice of exclusive breastfeeding with females being more likely to experience this practice.

**Table 2:** Comparison of feeding practices by gender for underfives

Variable	*N*	Female (%)	Male (%)	*p*
Exclusive breastfeeding				
Yes	70	29.4	5.6	0.00
No	366	70.6	94.6	
Age child started other foods/fluids				
1–3 months	181	39.2	43.4	0.00
4–5 months	185	31.4	51.2	
At 6 months (exclusively breastfed)	70	29.4	5.4	
Types of food/fluid child started				
Water	84	17.0	21.1	0.5
Cow's milk	167	41.8	35.5	
Plain thin porridge	150	33.0	35.5	
Kideri^a^	35	8.2	7.9	
Daily food frequency				
Two times	41	41.5	58.5	0.3
Three times	166	47.0	53.0	
Four times	191	45.5	54.5	
Five times	38	31.6	68.4	

a Banana mixed with sour milk.

### 3.2 Prevalence of diseases

The prevalence of morbidity was 67.4% (*n* = 294), with the most commonly reported diseases being diarrhea, fever, and cough. About 67% (*n* = 194) of children aged 12–24 months were reported to be ill. Diarrhea and multiple symptoms (i.e., diarrhea, fever, and/or cough) were prevalent among children aged 6–24 months, reported as 29% (*n* = 56) and 50.8% (*n* = 98), respectively (*p* < 0.001).

### 3.3 Nutritional status

Table 3 summarizes the comparison of prevalence and distribution of child undernutrition and clearly highlights that the majority of children, and males, specifically, exhibited at least one type of undernutrition.

**Table 3:** Comparison of Prevalence and distribution of child undernutrition by gender

Variable	*N*	Female (%)	Male (%)	*p*
Undernutrition	271	56.2	66.9	0.02
Underweight	123	23.2	32.2	0.04
Stunted	218	43.8	55.0	0.00
Wasted	72	12.9	19.4	0.06

### 3.4 Factors associated with underweight, stunting, and wasting

Table 4 shows the determinants of underweight among children aged 6–59 months. In univariate logistic regression, four determinants were significantly associated with being underweight. These were being a male (OR: 1.4, 95% CI: 1.0–2.3), age above 2 years (OR: 1.7, 95% CI: 1.1–2.7), being ill within the 2 weeks prior to data collection (OR: 4.5, 95% CI: 2.5–8.0), and living in Oturumeti ward (OR: 1.5, 95% CI: 2.6–7.8) or Seliani ward (OR 1.5: 95% CI: 0.8–2.6). In multivariate logistic regression, only three risk determinants were significantly associated with being underweight, namely child age above 2 years (AOR: 1.7, 95% CI: 1.02–2.7), recent illness (AOR: 4.2, 95% CI: 2.3–7.6), and living in Oturumeti (AOR: 4.6, 95% CI: 2.6, 8.2) or Seliani (AOR: 1.9, 95% CI: 1.1–3.5).

**Table 4:** Determinants of underweight among children under the age of 5 years

Variable	*N* (%)	COR (95% CI)	*p*‐Value	AOR (95% CI)	*p*‐Value
Child's sex					
Female	194 (23.2)	1		1	
Male	242 (32.2)	1.6 (1.0–2.3)	0.04	1.2 (0.7–1.9)	0.5
Child's age (months)					
6–24	291 (24.4)	1		1	
Above 24	145 (35.9)	1.7 (1.1–2.7)	0.01	1.7 (1.02–2.7)	0.04
Illness weak preceding survey					
No	142 (11.3)	1		1	
Yes	294 (36.4)	4.5 (2.5–8.0)	0.00	4.2 (2.3–7.6)	0.00
Exclusive breastfeeding					
Yes	70 (15.7)	1		1	
No	366 (30.6)	2.4 (1.2–4.7)	0.01	2.8 (1.3–5.8)	0.00
Residence					
Oldonyosambu	159 (17.0)	1		1	
Seliani	150 (23.3)	1.5 (0.8–2.6)	0.2	1.9 (1.1–3.5)	0.03
Oturumeti	127 (48.0)	4.5 (2.6–7.8)	0.00	4.6 (2.6–8.2)	0.00

*Note*

AOR, adjusted odds ratio.

The determinants of stunting are summarized in Table 5. Many factors were associated with stunting whereby three risk factors were significantly linked with stunted. These were being a male (OR: 1.6, 95% CI: 1.1–2.3), child age above 2 years (OR: 1.6 95% CI: 1.1–2.4), and being ill in the past 2 weeks prior to the survey (OR: 1.5, 95% CI: 0.9–2.2), having a father with primary education (OR: 0.4, 95% CI: 0.2–0.8), and living in Oturumeti (OR 1.7, 95% CI: 1.1–2.7). Multivariate logistic regression indicated that two determinants were significantly linked with stunting namely being a male (AOR: 1.5, 95% CI: 1.1–2.4) and age above 2 years (AOR: 1.7, 95% CI: 1.1–2.5).

**Table 5:** Determinants of stunting among children under the age of 5 years

Variable	*N* (%)	COR (95% CI)	*p*‐Value	AOR (95% CI)	*p*‐Value
Child's gender					
Female	194 (43.8)	1		1	
Male	242 (55.0)	1.6 (1.12.3)	0.02	1.6 (1.1–2.4)	0.01
Child's age (months)					
6–24	291 (46.0)	1		1	
Above 24	145 (57.9)	1.6 (1.1–2.4)	0.02	1.7 (1.1–2.5)	0.01
Illness weak preceding survey					
No	142 (43.7)	1			
Yes	294 (53.1)	1.5 (0.9–2.2)	0.06		
Exclusive breastfeeding					
Yes	70 (44.3)	1			
No	366 (85.8)	1.3 (0.8–2.2)	0.3		
Father's education					
None or primary incomplete	36 (69.4)	1			
Primary complete	247 (47.8)	0.4 (0.2–0.8)	0.02		
Secondary and above	53 (50.9)	0.4 (0.2–1.0)	0.08		
Residential area					
Oldonyosambu	159 (45.9)	1		1	
Seliani	150 (46.7)	1.0 (0.7–1.6)	0.8	1.1 (0.7–1.7)	0.7
Oturumeti	127 (59.1)	1.7 (1.1–2.7)	0.02	1.6 (0.9–2.6)	0.06

*Note*

AOR, adjusted odds ratio.

The determinants of wasting are summarized in Table 6. In univariate logistic regression, factors which were significantly associated with wasting were being ill a week before survey (OR: 3.5: 95% CI: 1.8–7.0), not exclusively breastfed (OR: 2.3: 95% CI: 0.9–5.6), having mother aged 35 years and above (OR: 2.6: 95% CI: 1.1–6.3), and living in Oturumeti (OR: 5.2: 95% CI: 2.6–10.2). In multivariate logistic regression, factors that remained significantly associated with wasting were being ill in the 2 weeks prior to the survey (AOR: 3.1, 95% CI: 1.5–6.4), children not exclusively breastfed (AOR: 2.5, 95% CI: 1.0–6.3), children born to mothers above 35 years (AOR: 3.9, 95% CI: 1.5, 10.6), and living in Oturumeti (AOR: 5.9, 95% CI: 2.9–12.2).

## 4 DISCUSSION

Our study indicates that almost half of underfive children in the study population were undernourished and that this group was disproportionately represented by males. This study showed a prevalence of stunting (50%), underweight (28.20%), and wasting (16.5%). Child age above 2 years, being a male, morbidity, nonexclusive breastfeeding, maternal age above 35 years, and living at Oturumeti or Seliani were predictors of children being undernourished.

**Table 6:** Determinants of wasting among children under the age of 5 years

Variable	*N* (%)	COR (95% CI)	*p*‐Value	AOR (95% CI)	*p*‐Value
Child's gender					
Female	194 (12.9)	1		1	
Male	242 (19.4)	1.6 (0.97–2.7)	0.06	1.1 (0.6–2.0)	0.7
Child's age (months)					
6–24	291 (15.1)	1			
Above 24	145 (19.3)	1.3 (0.8–2.3)	0.2		
Illness weak preceding survey					
No	142 (7.0)	1		1	
Yes	294 (21.1)	3.5 (1.8–7.0)	0.00	3.1 (1.5–6.4)	0.00
Exclusive breastfeeding					
Yes	70 (8.6)	1		1	
No	366 (18.0)	2.3 (0.9–5.6)	0.05	2.5 (1.0–6.3)	0.05
Mother's age					
15–24	213 (16.1)	1		1	
25–34	197 (13.7)	0.8 (0.5–1.3)	0.4	0.9 (0.5–1.6)	0.6
35–49	26 (34.6)	2.6 (1.1–6.3)	0.03	3.9 (1.5–10.6)	0.00
Residential area					
Oldonyosambu	159 (8.2)	1		1	
Seliani	150 (12.7)	1.6 (0.7–3.4)	0.2	1.9 (0.9–4.0)	0.1
Oturumeti	127 (31.5)	5.2 (2.6–10.2)	0.00	5.9 (2.9–12.2)	0.00

*Note*

AOR, adjusted odds ratio.

The research shows stunting is a significant health problem among infants and young children in Tanzania (Kulwa, Mamiro, Kimanya, Mziray, & Kolsteren, 2015; Safari, Kimambo, & Lwelamira, 2013). Stunting affected 55% of male children which is twice as high as the reported regional prevalence of stunting (27%) reported by Tanzania National Nutrition Survey (TFNC, 2014) and slightly higher than the 36% prevalence reported in the TDHS (National Bureau of Statistics & Macro, 2015). Likewise, underweight and wasting remain at unacceptably high levels, with study findings exceeding those of the regional reports (National Bureau of Statistics & Macro, 2015; Tanzania Food and Nutrition Centre, 2014). Rural areas account for the largest number of stunted children in the current study, which mirrors previous reports (Mamiro et al., 2005; National Bureau of Statistics & MACRO, 2010, 2015; Shirima et al., 2015). UNICEF et al. (2015) regard stunting as “very high” if it exceeds 40% in a population. This threshold was exceeded in our study population, which highlights the need for more urgent efforts to tackle the problem. Other studies reported stunting causative factors include poor maternal nutrition which may contribute to intrauterine growth retardation, preterm and low birthweight babies, inadequate complementary feeding, and frequent infections (Black et al., 2008; Prendergast et al., 2014).

As previously stated, male children were more likely to be stunted than their female counterparts, which mirrors findings described previously (Agho et al., 2009; Asfaw, Wondaferash, Taha, & Dube, 2015; Chirande et al., 2015; Hien & Hoa, 2009; Vitolo, Gama, Bortolini, Campagnolo, & Drachler Mde, 2008; Wamani, Åstrøm, Peterson, Tumwine, & Tylleskär, 2007). Compared with their female cohort, the likelihood of underweight was higher among males when not adjusted, but nonsignificant when adjusted for other factors. The higher odds of stunting in male children may be attributed to the early introduction of complementary feeds, with most (95%) males versus many (70%) of females introduced to complementary feeding before the WHOs (2018) recommended age. Early introduction of complementary feeding also may expose children to infections including acute respiratory infections, diarrhea, and worm manifestation (Arifeen et al., 2001; Kalanda, Verhoeff, & Brabin, 2006). Future studies are needed to consider gender‐specific and gender‐neutral approaches to address this issue of complementary feeding patterns.

The increased paternal educational level was shown to reduce the risk of child stunting. This finding is consistent with other studies conducted in Africa which suggested paternal education to be a protective factor against childhood stunting (Agho et al., 2009; Amsalu & Tigabu, 2008; Wamani, Tylleskär, Åstrøm, Tumwine, & Peterson, 2004). The rationale attributed to this linkage appears to align with the increased economic power to provide quality food and services for the family (Islam et al., 2013), as in most developing contexts the presence of a father contributes to the economic power of the family.

The findings suggested that the risk of undernutrition increases with age; specifically, children above 2 years were found to be at greater risk of being underweight and stunted. Similar findings were reported by other researchers (Ergin, Okyay, Atasoylu, & Beser, 2007; Olack et al., 2011; Vitolo et al., 2008). This finding may be attributed to poor child care and feeding practices where parents leave the child to be more independent. Also, at this age, some mothers might be pregnant or have another baby necessitating attending to the needs of the newborn and potentially neglecting the older one (Buitrón, Hurtig, & San Sebastián, 2004). Beyond 2 years, stunting is irreversible, so significant efforts must be focused on the first 1,000 days of life.

Childhood illness over the previous 14 days was associated with underweight and wasting (Basit, Nair, Chakraborthy, Darshan, & Kamath, 2012; Manyike et al., 2014). Infections impair the immune competence to fight against diseases. Illness causes failure to gain weight or loss of weight due to increase in energy expenditure, demand for nutrients, and depressed appetite (Asfaw et al., 2015). In this study, diarrhea and multiple infections were more prevalent among children aged 6–23 months. These patterns seem to align with the introduction of complementary foods, which may be contaminated due to the preparation and hygienic issues.

Maternal age above 35 years was identified as a risk factor for wasting among children. This finding is contrary to other studies which showed that wasting was more prevalent among children born to young mothers (<20 years) (Chirande et al., 2015). This variance may lead to an interesting consideration of the two age extremes and what each grouping might bring to the child's nutritional status.

Living at Oturumeti or Seliani were risk factors for underweight and/or wasted status, which may be related to cultural behaviors and climatic conditions which may contribute to the differential pattern from children in Oldonyosambu. One element may relate to gender‐based roles such as child care and household chores as primarily a woman's responsibility, whereas resources are controlled by men which may lead to conflicting priorities leading to child undernutrition. This type of variance is also asserted by other studies (Ergin et al., 2007; Hien & Hoa, 2009).

## 5 CONCLUSION

In conclusion, the high prevalence rates of undernutrition among children below the age of 5 years were observed in selected communities within Arusha District. Male children were found to be at greater risk of being malnourished than female counterparts, which was evidenced by the presence of stunting in nearly half of all male children. Through factor analysis, factors associated with child undernutrition in this study were as follows: being male, child age above 2 years, nonexclusive breastfed children, being born to a mother of 35 years and above, morbidity within the 2 weeks prior to the study, and area of residence (specifically, rural areas of Oturumeti and Seliani).

It is recommended from this study that nutritionists and health planners should focus on these key predictors when planning nutrition interventions to address the problem of undernutrition among underfive children in Arusha District. Future studies are needed to consider gender‐specific and gender‐neutral approaches to address the issue of complementary feeding patterns.

## CONFLICT OF INTERESTS

The authors declare that they have no competing interests.
